# Different gap junction-propagated effects on cisplatin transfer result in opposite responses to cisplatin in normal cells versus tumor cells

**DOI:** 10.1038/srep12563

**Published:** 2015-07-28

**Authors:** Yuan Zhang, Liang Tao, Lixia Fan, Yuexia Peng, Kefan Yang, Yifan Zhao, Qi Song, Qin Wang

**Affiliations:** 1 Department of Pharmacology, Zhongshan School of Medicine, Sun Yat-Sen University, Guangzhou 510080, People’s Republic of China

## Abstract

Previous work has shown that gap junction intercellular communication (GJIC) enhances cisplatin (Pt) toxicity in testicular tumor cells but decreases it in non-tumor testicular cells. In this study, these different GJIC-propagated effects were demonstrated in tumor versus non-tumor cells from other organ tissues (liver and lung). The downregulation of GJIC by several different manipulations (no cell contact, pharmacological inhibition, and siRNA suppression) decreased Pt toxicity in tumor cells but enhanced it in non-tumor cells. The *in vivo* results using xenograft tumor models were consistent with those from the above-mentioned cells. To better understand the mechanism(s) involved, we studied the effects of GJIC on Pt accumulation in tumor and non-tumor cells from the liver and lung. The intracellular Pt and DNA-Pt adduct contents clearly increased in non-tumor cells but decreased in tumor cells when GJIC was downregulated. Further analysis indicated that the opposite effects of GJIC on Pt accumulation in normal versus tumor cells from the liver were due to its different effects on copper transporter1 and multidrug resistance-associated protein2, membrane transporters attributed to intracellular Pt transfer. Thus, GJIC protects normal organs from cisplatin toxicity while enhancing it in tumor cells *via* its different effects on intracellular Pt transfer.

Gap junctions (GJs) are plasma membrane channels that mediate direct cell-to-cell transfer of cytoplasmic signaling molecules such as cyclic AMP, cyclic GMP, nucleotides, amino acids and glutathione^1^. GJ are formed of two hemichannels, each of which contains six connexin (Cx) monomers and docks to its counterpart in neighboring cells to form a gap junction channel^2^. Gap junction intercellular communication (GJIC) is crucial in diverse processes, including normal and pathological physiology, differentiation, development and cell death^3^. Likewise, accumulating evidence has suggested that GJ-mediated intercellular communication is of considerable value in cancer biology and its therapeutic utility^4^,^5^.

GJIC is frequently reduced or absent in cancer cells compared to their original tissue because of reduced Cx expression and/or aberrant localization of Cx proteins^6^. However, some cancers still retain significant GJIC^4^,^5^,^7^, and GJIC upregulation in nominally GJIC-deficient cancers can be detected during cancer progression (*e.g.*, during the transition to a metastatic phenotype)^8^,^9^. Accumulating evidence has suggested that GJIC enhances the toxicity of chemotherapeutic agents and radiotherapy in cancer cells^10^,^11^,^12^,^13^,^14^. This enhanced toxicity is due to intercellular diffusion of lethal signals through GJ channels. Therefore, GJs are considered potential targets for enhancing cancer therapies^15^. Nevertheless, because GJIC is generally more abundant among normal cells than among cancer cells, the lethal signals transferred by GJIC and produced by anti-tumor agents could be even greater among normal cells than among cancer cells, which would be therapeutically counterproductive. However, GJIC has been reported to mediate protective effects against cellular stress in normal cells^16^,^17^,^18^. Our previous study demonstrated that the effect of GJIC on cisplatin toxicity differs between normal and tumor testicular cells: GJIC protects normal cells from cisplatin toxicity while enhancing it in tumor cells, suggesting that the enhancement/maintenance of GJIC increases therapeutic efficacy while decreasing off-target toxicity^19^. However, whether these opposite effects of GJIC on normal and tumor testicular cells exist in cells from other organs and the exact mechanisms of the effects remain unclear.

In the present study, we confirmed these different GJIC-propagated effects on tumor versus non-tumor cells from other organ tissues (liver and lung). GJIC downregulation decreased Pt toxicity in tumor cells but enhanced it in non-tumor cells. We found that the intracellular Pt and the DNA-Pt adduct contents were clearly increased in non-tumor cells but decreased in tumor cells when GJIC was inhibited. Further analysis indicated that the opposite effects of GJIC on Pt accumulation in normal versus tumor cells from liver were due to its different effects on copper transporter 1 and multidrug resistance-associated protein 2, the membrane transporters attributed to intracellular Pt transfer. Thus, GJIC protects normal organs from cisplatin toxicity while enhancing it in tumor cells *via* its different effects on intracellular Pt transfer.

## Results

### Different effects of cell density on cisplatin toxicity in tumor and non-tumor cells

**Figure 1:**
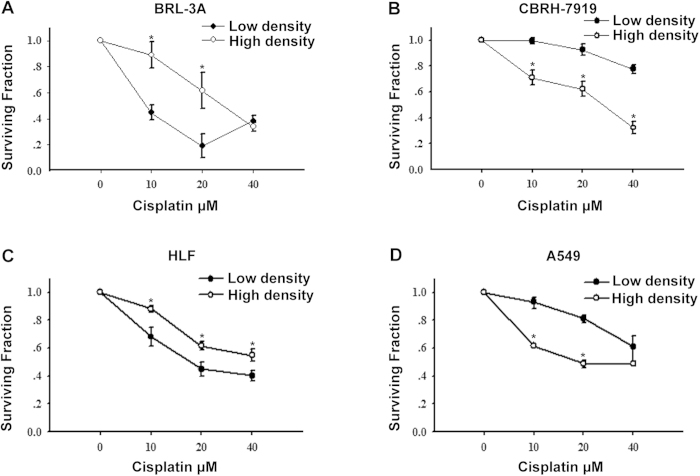
Clonogenic survival of liver (BRL-3A and CBRH-7919 cells) and lung cells (HLF and A549 cells) in response to cisplatin treatment. Standard colony formation assay was performed in (**A**) BRL-3A, (**B**) CBRH-7919, (**C**) HLF and (**D**) A549 cells incubated for 1 h with increasing doses of cisplatin under both high- and low-density culture conditions. The results are expressed as the mean ± s.e.m. (four to eight experiments); *P < 0.05, differs significantly from low-density culture conditions.

As an initial experiment to determine the effect of GJIC on cisplatin cytotoxicity, cells were cultured separately in 6-well plates under two conditions: a low-density condition where GJ formation was not possible and a high-density condition that allowed cells to come into contact with each other to form GJs. Standard colony formation assay revealed that all cisplatin concentrations reduced cell survival under both culture conditions. However, the effects of cell density were opposite in tumor and non-tumor cells. Under the high-density condition, the survival rate of non-tumor cells (BRL-3A and HLF cells) was substantially greater in response to cisplatin. In contrast, cell viability was considerably less in tumor cells (CBRH-7919 and A549 cells). As shown in Fig. 1, after the cells were exposed to 20 μM cisplatin for 1 h, the colony formation ability of non-tumor cells (BRL-3A and HLF cells) increased by 43% and 17% under the high-density condition compared to that under the low-density condition, respectively. In contrast, cell survival of tumor cells (CBRH-7919 and A549 cells) was reduced by 31% and 32% under the high-density condition compared to cultures under the low-density condition, respectively.

### Effects of cell density are mediated by GJIC

The cell density-dependent cisplatin sensitivity in connexin-expressing cells suggests a possible role for intercellular communication mediated by GJs. To examine the role of GJIC in cisplatin sensitivity, GJ function was manipulated by two methods: pharmacological inhibition of junctional channels and specific knockdown of dominant Cx expression by siRNA.

**Figure 2:**
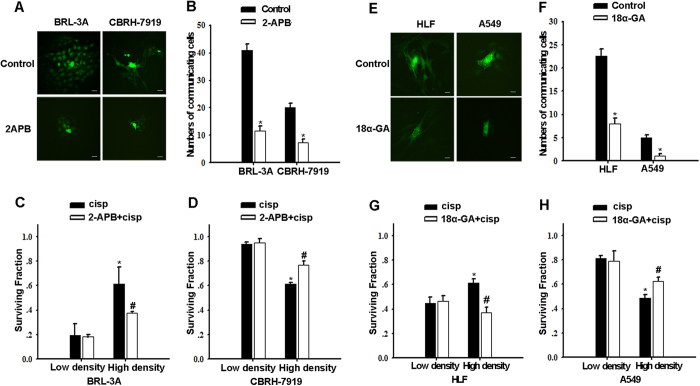
Effect of 2-APB and 18α-GA on cisplatin cytotoxicity in liver and lung cells. (**A,B,E,F**) ‘Parachute’ assay was used to determine the degree of GJIC of liver (BRL-3A and CBRH-7919 cells) and lung cells (HLF and A549 cells). Bars, s.e.m.; *P < 0.05, differs significantly from control (three to six independent experiments). The scale bars represent 20 μm. (**C,D,G,H**) Clonogenic survival of liver cells (BRL-3A and CBRH-7919 cells) and lung cells (HLF and A549 cells) incubated with 20 μM cisplatin for 1 h with or without 50 μM 2-APB or 50 μM 18α-GA treatment under both high- and low-density culture conditions. The results are expressed as the mean ± s.e.m. (four to eight experiments). Bars, s.e.m. *P < 0.05, differs significantly from the low-density group; #P < 0.05, differs significantly from cisplatin.

Treatment of BRL-3A and CBRH-7919 cells with 2-APB (50 μM), which specifically inhibits hemichannels that are unpaired and junctional channels that are composed of Cx32^20^, blocked the spread of calcein through Cx32 GJs (Fig. 2A,B). Under the low-density condition, 2-APB pretreatment did not affect cisplatin cytotoxicity (Fig. 2C,D). However, under the high-density condition, 2-APB pretreatment of non-tumor cells (BRL-3A cells) that were exposed to cisplatin dramatically reduced the survival rate from 0.62 ± 0.14 to 0.38 ± 0.01 (Fig. 2C), whereas 2-APB pretreatment of tumor cells (CBRH-7919 cells) that were exposed to cisplatin increased the survival rate from 0.62 ± 0.06 to 0.77 ± 0.03 (*P *< 0.05) (Fig. 2D). Similarly, in HLF and A549 cells that were exposed to cisplatin under the high-density condition, treatment with the GJ inhibitor 18α-GA^21^ decreased the cell survival rate from 0.61 ± 0.03 to 0.44 ± 0.02 in non-tumor cells (HLF cells) but increased it from 0.49 ± 0.02 to 0.62 ± 0.03 in tumor cells (A549 cells). In contrast, GJIC inhibitor treatment hardly affected the cell survival rate after cisplatin exposure under the low-density condition (Fig. 2E–H).

**Figure 3:**
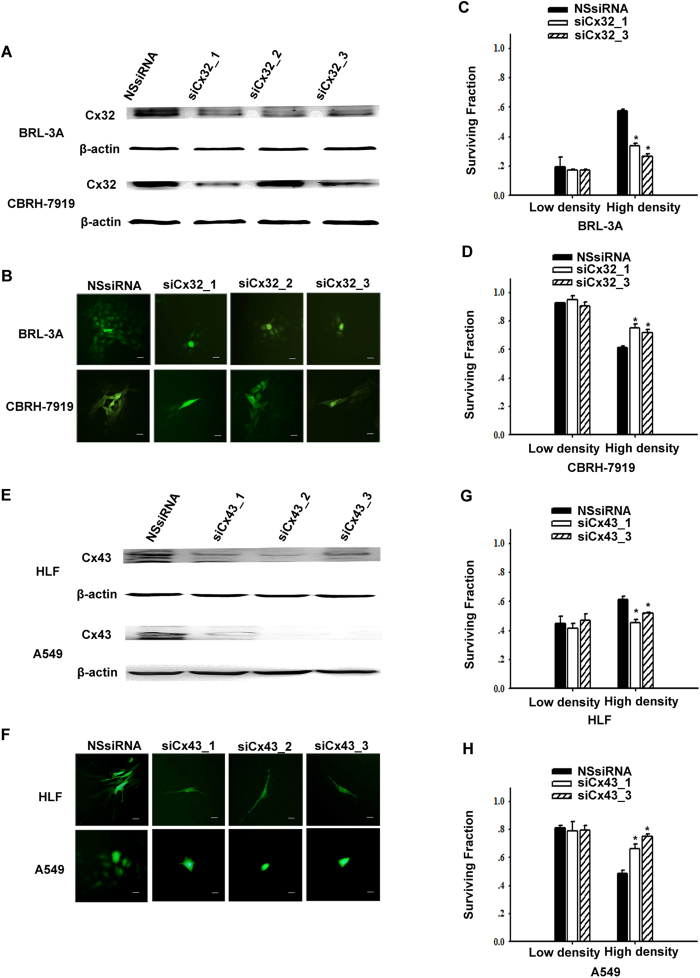
Effect of siRNA-mediated knockdown of connexin expression on cisplatin cytotoxicity. (**A,B**) Western blotting and parachute assay were used to assess Cx32 expression and GJIC respectively after transfection of Cx32-siRNA in BRL-3A and CBRH-7919 cells. The scale bars represent 20 μm. (**C,D**) Clonogenic survival of non-specific siRNA (NSsiRNA) and Cx32-siRNA-transfected liver cells (BRL-3A and CBRH-7919 cells) after 1 h of incubation with 20 μM cisplatin. The results are expressed as the mean ± s.e.m. (three to seven experiments); *P < 0.05, differs significantly from NSsiRNA. (E, F) Western blotting and parachute assay were used to assess Cx43 expression and GJIC respectively after transfection of Cx43-siRNA in HLF and A549 cells. The scale bars represent 20 μm. (**G,H**) Surviving fractions of NSsiRNA- and Cx43-siRNA-transfected lung cells (HLF and A549 cells) after 1 h of incubation with 20 μM cisplatin. The results are represented as the mean ± s.e.m. (three to six experiments); *P < 0.05, differs significantly from NSsiRNA.

To further assess the role of GJIC in cisplatin cytotoxicity, siRNA transfection was applied to knockdown Cx32 or Cx43 expression. Cx32 or Cx43 knockdown did not influence cisplatin toxicity in low-density cultures of all cell types. In contrast, in high-density cultures, siRNA transfection decreased the surviving fraction in non-tumor cells (BRL-3A and HLF cells) and increased it in tumor cells (CBRH-7919 and A549 cells) (Fig. 3).

Taken together, these experiments clearly demonstrated that inhibiting GJIC composed of Cx32 or Cx43 by either chemical inhibitor inhibition or Cxs knockdown had essentially the same effects on cisplatin toxicity as did cell density. The opposite GJIC-mediated effect was reflected in protection against cisplatin toxicity in non-tumor cells but was enhanced in tumor cells.

### *In vivo* assay of different GJ-mediated effects on cisplatin toxicity

**Figure 4:**
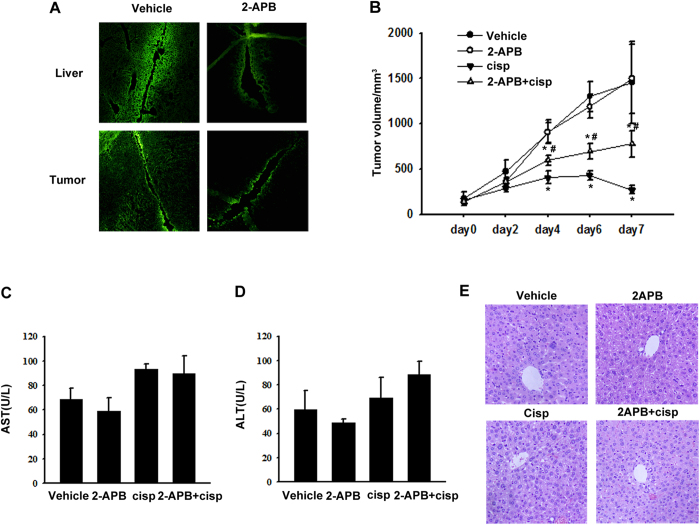
Effect of GJIC on tumor xenograft growth and cisplatin-induced hepatoxicity. (**A**) Tissue GJIC was evaluated by scrape-loading/dye transfer assay in mice liver and tumor. (**B**) Volume of tumors in each treatment group. *P < 0.05, differs significantly from the vehicle group; #*P* < 0.05, differs significantly from the cisplatin group (n = 10). (**C,D**) Serum AST and ALT levels (**E**) and HE liver histology (n = 10). The scale bars represent 20 μm.

For *in vivo* assessment, a xenograft tumor model of transplanted CBRH-7919 cells was applied. Mice were administered 20% DMSO or 20 mg kg^−1^ 2-APB, and then a scrape-loading/dye transfer assay was performed. A decrease in Lucifer Yellow spread was observed in liver and tumor tissues from 2-APB-treated mice, indicating that 2-APB effectively blocked GJIC in liver and tumor tissues *in vivo* (Fig. 4A).

The hypothesis that GJIC enhanced the antitumor effect of cisplatin while protecting normal liver cells was examined *in vivo*. Compared to the control, tumor progression was significantly retarded in the mice treated with 5 mg kg^−1^ cisplatin alone but was less retarded in those treated with cisplatin and 2-APB. 2-APB itself did not affect tumor progression (Fig. 4B). These results indicated that GJIC enhanced the antitumor effect of cisplatin. However, the serum levels of AST and ALT in all groups of mice did not differ significantly, and no histological damage was observed in the liver tissues (Fig. 4C–E). All these findings suggested that cisplatin did not cause obvious hepatotoxicity at the therapeutic dose.

### Different effects of GJIC on Pt uptake in tumor and non-tumor cells

**Figure 5:**
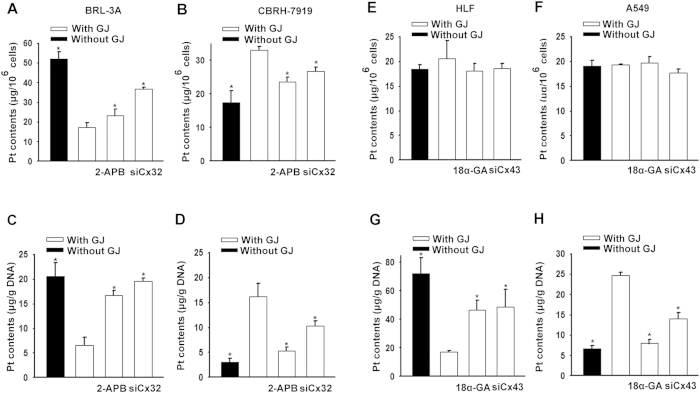
Effects of GJIC on Pt uptake in liver (BRL-3A and CBRH-7919 cells) and lung cells (HLF and A549 cells). Intracellular Pt content (**A,B,E,F**) and DNA-Pt adduct contents (**C,D,G,H**) were assessed in liver (BRL-3A and CBRH-7919 cells) and lung cells (HLF and A549 cells) after 1 h of incubation with 20 μM cisplatin with and without GJs. The results are expressed as the mean ± s.e.m. (four to ten experiments); *P < 0.05, differs significantly from the cisplatin treatment group with GJ.

The intracellular Pt content was measured at 1 h after exposure to 20 μM cisplatin and was normalized as Pt μg/10^6^ cells^22^. In non-tumor cells (BRL-3A cells), the level of cellular Pt increased when GJ was blocked by the following manipulations: low-density culture, 2-APB treatment, and Cx32-siRNA transfection. In contrast, the Pt content in tumor cells (CBRH-7919 cells) was reduced by GJIC inhibition (Table 1; Fig. 5A,B). Thus, in the cells with GJs composed of Cx32, the changes in cisplatin toxicity could be partly attributed to GJIC-mediated alterations of Pt uptake.

Next, we measured the DNA-bound Pt content in the cells at 1 h after exposure to 20 μM cisplatin. The DNA-Pt content was normalized as Pt μg/gDNA^23^. DNA-Pt adduct content increased in BRL-3A cells but decreased in CBRH-7919 cells when GJIC was inhibited by the three manipulations described above (Table 1; Fig. 5C,D), suggesting that the changes in Pt accumulation in cells were accompanied by a proportional increase or decrease in the amount of cisplatin-induced DNA inter-strand crosslinks.

In addition, in non-tumor cells and tumor cells from lung tissue (A549 and HLF cells), Pt accumulation in cells did not alter when GJIC was blocked. However, the DNA-Pt adduct contents decreased in A549 cells but increased in HLF cells after inhibition of GJIC (Table 1; Fig. 5E–H).

### GJIC affects Pt transfer *via* CTR1 or MRP2

**Figure 6:**
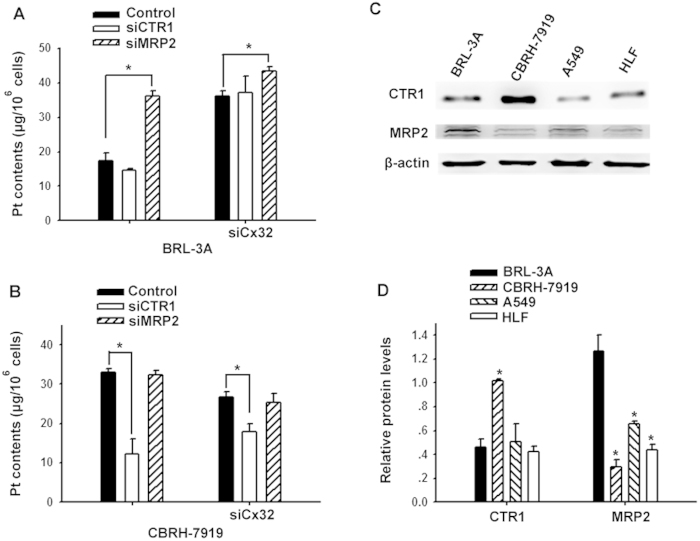
GJIC-mediated cisplatin transfer was related to CTR1 and MRP2. (**A,B**) Intracellular Pt content was measured when transfected with CTR1-siRNA and MRP2-siRNA both with and without GJ in liver cells (BRL-3A and CBRH-7919 cells). The results are represented as the mean ± s.e.m. (three to five experiments). *P < 0.05, differs significantly from control group. (**C,D**) Expression of CTR1 and MRP2 in BRL-3A, CBRH-7919, A549 and HLF cells. The results are represented as the mean ± s.e.m. (three experiments). *P < 0.05, differs significantly from expression in BRL-3A cells.

To explore the mechanisms underlying the different effects of GJIC on Pt transfer in tumor and non-tumor cells, we investigated the role of Pt transfer-related transporters in the effects of GJIC. Several active transporters are related to intracellular Pt transfer including influx transporters (copper transporter 1, CTR1) that transport cisplatin from extracellular fluid into the cells and efflux transporters (multidrug resistance-associated protein 2, MRP2) that transport cisplatin out of the cells. Figure 6C,D showed that both hepatocytes (BRL-3A) and hepatoma cells (CBRH-7919) expressed CTR1 and MRP2. The level of CTR1 expression was higher in tumor cells than that in non-tumor cells. In contrast, the expression level of MRP2 was higher in non-tumor cells than that in tumor cells. CTR1-siRNA or MRP2-siRNA was transfected into BRL-3A and CBRH-7919 cells, respectively, to knockdown CTR1 or MRP2 expression (Supplementary Fig. S1). In non-tumor cells (BRL-3A), CTR1 knockdown did not change intracellular Pt accumulation, indicating that CTR1 was not related to Pt uptake in non-tumor cells. However, MRP2 knockdown increased the intracellular Pt content by 76% in the cells with GJs and by 42% in the cells in which GJs were inhibited by using Cx32-siRNA (Fig. 6A). These results demonstrated that MRP2 participated in cisplatin transfer in non-tumor hepatocytes and that GJ augmented the MRP2-mediated cisplatin efflux. In hepatoma cells (CBRH-7919), MRP2 knockdown did not affect the intracellular Pt content, suggesting that MRP2 was not related to Pt transfer in tumor cells. However, CTR1 knockdown reduced the intracellular Pt content by 63% in the cells with GJs or by 26% when cellular GJs were inhibited (Fig. 6B). These results indicated that CTR1 was involved in Pt transfer in the tumor cells and that GJ increased the CTR1-mediated cisplatin influx.

However, in a non-tumor cell line (HLF) and a tumor cell line (A549), both of which were derived from lung tissues, the levels of CTR1 and MRP2 expression were much lower compared to those in liver cells (Fig. 6C,D), suggesting that cisplatin transfer in lung cells occurred *via* different mechanisms.

**Figure 7:**
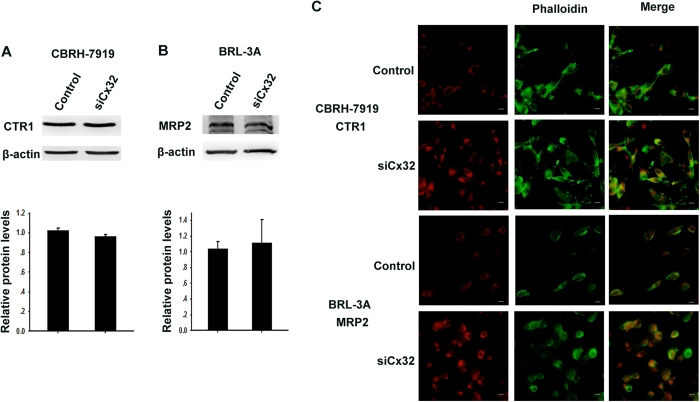
Effect of GJIC on CTR1 and MRP2 expression. (**A,B**) Western blotting assay was used to assess the expression of CTR1 and MRP2. (**C**) Localization of CTR1 and MRP2 in control and Cx32-siRNA-transfected cells by immunofluorescence. The scale bars represent 20 μm.

To investigate the mechanisms of the effect of GJIC on cisplatin transport, we examined the effect of GJIC on the expression and membrane localization of CTR1 or MRP2 in CBRH-7919 or BRL-3A cells. Knockdown of Cx32 did not alter the expression of CTR1 and MRP2 in these tumor and non-tumor cells (Fig. 7A,B). Immunofluorescence tests showed that CTR1 was localized predominantly in the plasma membrane of tumor cells, and knockdown of Cx32 strikingly increased the CTR1 in the cytoplasm. Similarly, in non-tumor cells (BRL-3A), MRP2 was localized predominantly in the plasma membrane, and knockdown of Cx32 increased the MRP2 in the cytoplasm (Fig. 7C).

## Discussion

In this study, we investigated the effects of GJIC on cisplatin cytotoxicity in normal versus tumor cells from liver and lung tissues. Our results show that GJIC enhances cisplatin cytotoxicity in tumor cells but reduces it in the corresponding normal cells, which demonstrates that the opposite effects of GJIC on cisplatin that we found previously in normal and tumor testicular cells also exist in cells from other origins with different oncogenic status. Although further studies are required to understand whether this finding is universal to other Cx-mediated GJIC and all chemotherapeutic agents, the work reported here suggests a key insight regarding intercellular signaling and bystander effects in response to chemotherapeutic and perhaps radiotherapeutic cancer treatments. Our results represent the first demonstration of a GJ-mediated protective effect on non-tumor cells upon exposure to a chemotherapeutic agent and suggest that targeted or transitory upregulation of GJIC is a profitable strategy to enhance the therapeutic efficiency of nonsurgical cancer treatments.

Cxs may affect cellular processes by GJIC-dependent and/or GJIC-independent mechanism(s). The former means that effect of Cxs is due to the formation of functional junctional channels. The GJIC-independent action of Cx is produced by the expression of the protein itself and/or by the formation of hemichannels. When GJs were not formed (in low-density cultures), the knockdown of either Cx32 or Cx43 by siRNA had no effect on cisplatin toxicity, indicating that Cx itself did not affect cisplatin toxicity. The treatment of cells without GJs with a GJIC inhibitor, 2-APB or 18α-GA, did not affect cisplatin toxicity, showing that hemichannels were not related to this effect. However, in all cells, inhibition of GJIC by chemicals or siRNAs reversed the effects of high-density culture on cisplatin toxicity, indicating that both modulatory effects of high-density culture were attributable to GJ formation and that GJIC differentially influences cisplatin cytotoxicity in normal and tumor cells.

The *in vivo* study results showed that 5 mg kg^−1^ cisplatin efficiently retarded xenograft tumor progression. This cisplatin-induced antitumor effect was reduced significantly by GJIC inhibition, which was consistent with the results from cell experiments, indicating that GJs function as tumor suppressors to restrict tumor growth. In the meantime, the therapeutic dose of cisplatin did not induce obvious increases in serum AST and ALT levels or histological damage, consistent with the report that the therapeutic dose of cisplatin did not cause detectable liver damage^24^. Together, these findings demonstrated that GJIC could potentiate the antitumor effect of cisplatin *in vivo* without enhancing hepatic damage.

Cisplatin enters cells primarily by passive diffusion and then binds to DNA in the nucleus, forming inter- and intra-strand crosslinks. These DNA crosslinks lead to the inhibition of replication and transcription and to the activation of downstream pathways that induce cell cycle arrest, DNA lesion repair and apoptosis or necrosis^25^. The GJIC-propagated signals (protective or toxic) that are responsible for the up- or downregulation of cisplatin cytotoxicity must be triggered by the above processes. In the present study, we observed that intracellular Pt accumulation increased in non-tumor cells (BRL-3A) and decreased in tumor cells (CBRH-7919) when GJIC was blocked, indicating that intracellular Pt accumulation might be modulated by GJIC. This finding suggests that GJIC-modulated Pt accumulation maybe responsible for the opposite effects of GJIC on cisplatin cytotoxicity in tumor versus non-tumor liver cells.

Active transporters in the uptake of cisplatin and other platinum compounds have been attributed to intercellular Pt accumulation^26^,^27^,^28^,^29^. A genetic screen of yeast and mouse cells demonstrated that the copper transporter CTR1 is a major determinant of cisplatin uptake and sensitivity^26^; this result has been confirmed in several different cell systems^28^,^30^,^31^,^32^. A copper chelator, tetrathiomolybdate (TM), enhances cisplatin uptake and cancer cell death in a CTR1-dependent manner without affecting normal organs in a mouse model of cervical cancer^33^. Furthermore, multidrug resistance-associated protein 2 (MRP2) could efflux glutathione conjugates of cisplatin from cancer cells, therefore conferring cytotoxicity resistance^34^,^35^,^36^ and protecting kidneys from cisplatin nephrotoxicity by reducing the burden of tubular cells^37^. Consequently, influx or efflux transporters of cisplatin are vital for cisplatin transfer in tumor and normal cells. This study is the first to demonstrate that GJIC significantly enhances CTR1-mediated Pt uptake in tumor cells (CBRH-7919) and MRP2-mediated efflux in non-tumor cells (BRL-3A). The reasons for this finding may be that CTR1 is highly expressed in CBRH-7919 cells, MRP2 has higher expression levels in BRL-3A cells, and the functions of both transporters could be enhanced by GJIC (Fig. 6). Therefore, the differential expression of CTR1 and MRP2 in tumor versus non-tumor cells possibly resulted in the opposite effects of GJIC on cisplatin transport and cytotoxicity.

However, as shown in Fig. 7A,B, knockdown of Cx32 did not alter CTR1 expression in CBRH-7919 cells or MRP2 expression in BRL-3A cells. To explore the mechanism underlying GJIC-induced intensification of CTR1 and MRP2-mediated cisplatin transfer in the case of invariant CTR1 and MRP2 expression, we examined the contents of CTR1 and MRP2 localized in the cell membrane. CTR1 and MRP2 are membrane proteins that determine cisplatin cytotoxicity^38^. The results of immunofluorescence test showed that the contents of either CTR1 or MRP2 significantly decreased in the cell membrane when Cx32 was knocked down (Fig. 7C), suggesting that GJIC was important for maintaining the level of CTR1 or MRP2 in the plasma membrane. Thus, GJ stabilized the membrane localization of active transporters and intensified the CTR1-mediated influx or MRP2-mediated efflux of cisplatin, subsequently decreasing cisplatin accumulation in non-tumor cells and increasing it in tumor cells.

The data present here indicate that the levels of CTR1 and MRP2 expression were much lower in the cells from lung tissues (A549 and HLF cells) than in the cells from liver tissues (CBRH-7919 and BRL-3A cells). Cisplatin may enter lung cells primarily by passive diffusion and may not depend on active transporters. In the cells originating from lung tissues (A549 and HLF cells), GJIC increased the DNA-adduct content in tumor cells and reduced it in non-tumor cells, while the intracellular cisplatin content did not changed, indicating that GJIC might affect the cisplatin-DNA adduct content by affecting the repair process of the platinum-DNA adduct. Evidence showed that GJs mediated the transfer of the DNA-dependent protein/Ku70/Ku80, which participates in the DNA repair system^39^. In addition, tightly coordinated mRNA expression of genes in the nucleotide excision repair pathway was demonstrated in normal brain cells, manifesting highly efficient DNA repair. Malignant brain tissues showed that the DNA repair efficiency was much lower, which was demonstrated by a reduced level of coordination of mRNA expression patterns for the same genes^40^,^41^. Therefore, GJIC might have enhanced DNA repair efficiency in normal cells (such as HLF) and then reduced cisplatin-DNA adducts. In contrast, GJIC decreased the DNA repair efficiency in tumor cells and then increased cisplatin-DNA adducts. Although further studies are required, the present study suggests that GJIC affects cisplatin cytotoxicity through its effects on intracellular Pt transfer or the DNA repair system, resulting in altered DNA-adduct contents.

## Methods

### Materials

Cisplatin, 2-aminoethoxydiphenyl-borate (2-APB), 18α-glycyrrhetinic acid (18α-GA), 70% nitric oxide, anti-Cx32, anti-Cx43, anti-β-actin mouse IgG, and secondary antibodies for western blot analysis were obtained from Sigma-Aldrich (St. Louis, MO, USA). Primary antibodies directed against CTR1 and MRP2 were purchased from Abcam (Cambridge, UK). Cell culture reagents, Lipofectamine™ 2000, calcein acetoxymethyl ester (calcein-AM), Lucifer Yellow, and secondary antibodies for immunofluorescence were obtained from Invitrogen (Carlsbad, CA, USA). Alexa Fluor® 488 Phalloidin was obtained from Cell Signaling Technology (Danvers, Massachusetts, USA). All other reagents were obtained from Sigma-Aldrich unless stated otherwise.

### Cell culture

A rat hepatocellular carcinoma cell line (CBRH-7919), rat liver cell line (BRL-3A) human non-small cell lung carcinoma cell line (A549) and human lung fibroblast cell line (HLF) were obtained from American Type Culture Collection (Manassas, VA, USA). The cells were cultured in Dulbecco’s modified Eagle’s medium supplemented with 10% fetal bovine serum at 37 °C in an atmosphere of 5% CO_2_ in air.

### Standard colony formation assay

Cisplatin was used at concentrations of 0–40 μM. Cells were exposed to cispaltin for 1 h in the dark. Cells were treated with 2-APB or 18α-GA at 50 μM for 1 h before incubation with cisplatin and remained during cisplatin treatment. Cisplatin toxicity was assessed by standard colony formation assay^11^. Cells were seeded at high density (30,000 cells/cm^2^), treated with cisplatin, then trypsinized and seeded into six-well dishes at 500 cells/cm^2^. For low density, cells were directly seeded at 500 cells/cm^2^ in six-well dishes and treated with cisplatin. Cells were incubated for another 5–10 days, then fixed and stained with 4% crystal violet in ethanol. Colonies containing 50 or more cells were scored. Colony formation was normalized to the colony forming efficiency of cells without cisplatin treatment.

### ‘Parachute’ dye coupling assay

GJ function was examined and performed as described by Goldberg^42^. Cells were grown to confluence in 12-well dishes. Donor cells were labeled with 5μM calcein-AM for 30 min at 37 °C and then rinsed, trypsinized and seeded onto the receiver cells at a 1:150 donor/receiver ratio. The cells were allowed to affix to the monolayer of receiver cells for 4 h at 37 °C and then observed using a fluorescence microscope (Olympus IX71, Tokyo, Japan). The average number of receiver cells containing calcein per donor cell was regarded as a measure of the degree of GJIC.

### siRNA transfection experiments

Cells were seeded and grown to 30%–50% confluence, and then the complexes of non-specific siRNA (NSsiRNA) or targeted siRNA (50 nM)(Ribbon, Guangzhou, China) and Lipofectamine™ 2000 were added to cells in each well according to the manufacturer’s procedure. Cells were incubated for another 48 h for colony assay. The sequences for the synthetic siRNAs targeting Cx32 (siCx32) were as follows: siCx32_1: 5′-CACCAACAACACATAGAAA-3′, siCx32_2: 5′-GCATCTGCATTATCCTCAA-3′, and siCx32_3: 5′-GCCTCTCACCTGAATACAA-3′. SiCx32_1 and siCx32_3 were chosen for further study. The sequences for the synthetic siRNAs targeting Cx43 (siCx43) were as follows: siCx43_1: 5′-GAACCTACATCATCAGTAT-3′, siCx43_2: 5′-CAGTCTGCCTTTCGTTGTA-3′, and siCx43_3: 5′-GGCTAATTACAGTGCAGAA-3′. SiCx43_2 and siCx43_3 were chosen for further study. The sequences for the synthetic siRNAs targeting CTR1 (siCTR1) were as follows: siCTR1_1: 5′-GGATGAACCACATGGAGAT-3′, siCTR1_2: 5′-GCTGCACATCATCCAAGTA-3′, and siCTR1_3: 5′-TCATCTTCATGACCTACAA-3′. The sequences for synthetic siRNAs targeting MRP2 (siMRP2) were as follows: siMRP2_1: 5′-GCAGGTGTTCGTCGTGTTT-3′, siMRP2_2: 5′-GGAGACGATTTAGACACAT-3′, and siMRP2_3: 5′-GCCTACAGCTTGGGTTGTT-3′. Knockdown of targeted protein expression was confirmed by western blot analysis.

### Western blot analysis

Whole-cell lysates were prepared in lysis buffer (20 mM Tris-HCl [pH7.4], 150 mM NaCl, 1 mM EDTA, 1 mM EGTA, 1% Triton X-100, 2.5 mM sodium pyrophosphate, 1 mM Na_3_VO_4_, 1 mM β-glycerophosphate, and 1:1,000 protease inhibitors), sonicated and centrifuged at 14,167 g for 30 min at 4 °C. Protein concentration was determined by using a DC protein assay kit (Bio-Rad Co., Hercules, CA, USA). In total, 25 μg of protein from each sample was loaded on a SDS–PAGE, separated by electrophoresis, and transferred to a nitrocellulose membrane. Membranes were blocked at room temperature for 1 h, followed by incubation with specific antibodies overnight at 4 °C. Monoclonal antibodies for Cx32 (1:1,000), Cx43 (1:3,000), CTR1 (1:1,000), MRP2 (1:100) and β-actin (1:10,000) were used. Immuno-positive bands were visualized using an Amersham ECL™ Plus Western Blotting Detection Kit (GE Healthcare, Piscataway, NJ, USA).

### Murine xenograft model system

Male athymic nude mice were used at 6–8 weeks of age (n = 40 mice; Guangdong Medical Lab Animal Center, China). The mice were housed in specific pathogen-free (SPF) conditions under a 12 h light-dark cycle and routinely fed basal rodent chow and water. All experiments were performed in accordance with the approved guidelines, and this study was approved by the ethics committee of Sun Yat-Sen University (Guangzhou, China). Each mouse was inoculated subcutaneously under the right flank with 3 × 10^6^ CBRH-7919 cells in PBS. Once the tumors had reached 100 mm^3^, the mice were divided randomly into four groups (10 mice per group) and treated intraperitoneally with vehicle (20%DMSO), 2-APB (20 mgkg^−1^), cisplatin (5 mgkg^−1^), or a combination of 2-APB and cisplatin every other day for a total four treatments. Tumor size was measured every other day with digital calipers and was calculated as V = 1/2 × (a × b^2^), where a is the largest superficial diameter and b is the smallest superficial diameter. Twelve hours after the mice received their final injections, they were anaesthetized and exsanguinated by abdominal aortic, and serum was obtained from blood samples. The serum alanine aminotransferase (ALT) and aspartate aminotransferase (AST) levels were measured to evaluate liver function^43^. Livers and tumors were excised from the mice immediately after blood collection, and then the livers were fixed in10% neutral formalin and embedded in paraffin for hematoxylin and eosin staining.

### Tissue scrape-loading/dye transfer assay for gap junction communication

*In vivo* GJIC was determined using scrape-loading/dye transfer assay^44^,^45^. Mice were injected intraperitoneally with vehicle (20% DMSO) or 2-APB (20 mg kg^−1^), and 3 h later, the liver and tumor were removed and freshly sliced. Incisions were made with a blade that was dipped into a solution of 0.5% Lucifer Yellow. Liver and tumor slices were incubated with the dye solution for 5 min, rinsed in saline, fixed in 4% paraformaldehyde for 30 min, embedded in OCT compound, sectioned at 10-μm thickness, and then observed using a fluorescence microscope.

### Measurement of Pt accumulation

Pt content was measured as describedpreviously^23^,^26^,^46^. In brief, cells were grown to confluence, treated with cisplatin, trypsinized and collected. For total cell platinum accumulation, the pellets were washed, suspended with PBS and 1% Triton X-100 in 0.1% SDS. For measuring the Pt content in DNA, DNA was harvested using a Wizard Genomic DNA Purification Kit (Promega, Madison, WI, USA) according to the manufacturer’s protocol. The total Pt and DNA-Pt contents were analyzed prior to the addition of 70% nitric acid using a Varian AA240Z atomic absorption spectrometer. Elemental platinum standards (5–100 μg L^−1^) were prepared by serial aqueous dilution using a high-purity platinum standard (1,000 μg mL^−1^). Each sample was measured twice, and concentrations were determined by applying an elemental platinum standard curve.

### Immunofluorescence

Cells were fixed within 4% paraformaldehyde and then blocked with 2% BSA. Then, CBRH-7919 and BRL-3A cells were incubated with primary antibody directed against CTR1 (1:400) or MRP2 (1:100), respectively, at 4 °C overnight, followed by incubation with FITC-conjugated goat anti-rabbit/mouse secondary antibody (1:400) for 1 h in the dark. Alexa Fluor® 488 Phalloidin was applied for 15 min to stain cytoarchitecture of cells. Then, the cells were washed with PBS and visualized using an Olympus IX71 fluorescence microscope.

### Statistical analysis

The data were statistically analyzed using one-way ANOVA and unpaired Student’s *t*-test. The data are presented as the mean ± standard error using Sigma Plot software (Jandel Scientific). *P* < 0.05 was considered significant.

## Additional Information

**How to cite this article**: Zhang, Y. *et al.* Different gap junction-propagated effects on cisplatin transfer result in opposite responses to cisplatin in normal cells versus tumor cells. *Sci. Rep.* **5**, 12563; doi: 10.1038/srep12563 (2015).

## Supplementary Material

Supplementary Information

## Figures and Tables

**Table 1:** Effects of GJIC on Pt accumulation in liver (BRL-3A and CBRH-7919 cells) and lung cells (HLF and A549 cells)

**Cell line**	**Whole cell Pt content**			**DNA Pt content**		
	**Without GJ**	**Inhibitor**	**siRNA**	**Without GJ**	**Inhibitor**	**siRNA**
BRL-3A	3.01 ± 0.18	1.35 ± 0.19	2.10 ± 0.07	3.14 ± 0.42	2.55 ± 0.15	2.98 ± 0.09
CBRH-7919	0.53 ± 0.10	0.71 ± 0.03	0.81 ± 0.03	0.19 ± 0.05	0.33 ± 0.05	0.64 ± 0.06
HLF	0.89 ± 0.04	0.88 ± 0.07	0.90 ± 0.05	4.18 ± 0.66	2.69 ± 0.41	2.83 ± 0.71
A549	0.99 ± 0.06	1.02 ± 0.06	0.92 ± 0.03	0.27 ± 0.02	0.32 ± ± 0.03	0.57 ± 0.05

Each value was ratio of Pt content compared to control cells containing GJ, and was represented as mean ± s.e.m. from three to eight independent experiments. *P* < 0.05.
