# Supplementary material for: Different gap junction-propagated effects on cisplatin transfer result in opposite responses to cisplatin in normal cells versus tumor cells
Source: Sci Rep. 2015 Jul 28;5:12563. doi: 10.1038/srep12563 (PMC4517168; doi:10.1038/srep12563)

Different gap junction-propagated effects on cisplatin transfer result in opposite responses to cisplatin in normal cells versus tumor cells

Yuan Zhang, Liang Tao, Lixia Fan, Yuexia Peng, Kefan Yang, Yifan Zhao, Qi Song, Qin Wang

Department of Pharmacology, Zhongshan School of Medicine, Sun Yat-Sen University, Guangzhou 510080, People's Republic of China

Running title: Gap junctions mediate cisplatin transfer

Note: Y.Z., and L.T. made equal contributions to this paper

Correspondence: Dr. Qin Wang, Department of Pharmacology, Zhongshan School of Medicine, Sun Yat-Sen University, Guangzhou, People's Republic of China, 510080. Tel & Fax: 86-20-87332318, Email: wangqin6@mail.sysu.edu.cn

Supplemental materials

Supplementary Figure S1: Assessment of siRNA-mediated knockdown of CTR1 and MRP2. (A) Western blotting was employed to assess levels of CTR1 after transfection of CTR1-siRNA in CBRH-7919 cell. (B) Western blotting was employed to assess

levels of MRP2 after transfection of MRP2-siRNA in BRL-3A cell.

Supplementary Figure S1

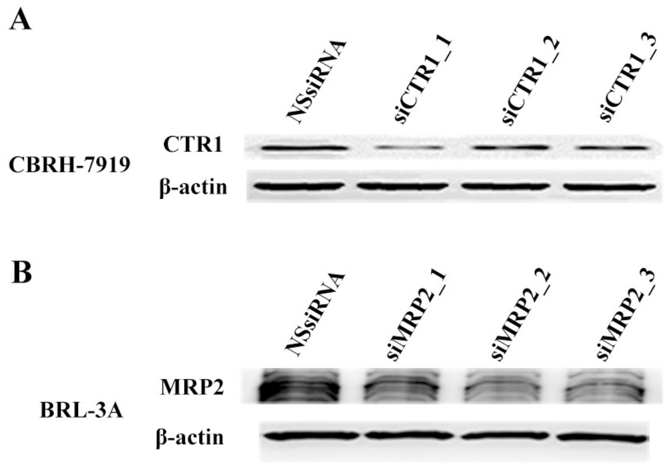

Supplement: Supplementary Information [file srep12563-s1.pdf]
